# Enhancement of Binding Kinetics on Affinity Substrates Using Asymmetric Electroosmotic Flow on a Sinusoidal Bipolar Electrode

**DOI:** 10.3390/mi13020207

**Published:** 2022-01-28

**Authors:** Yupan Wu, Bowen Hu, Xun Ma, Yucheng Wang, Wei Li, Shaoxi Wang

**Affiliations:** 1 School of Microelectronics, Northwestern Polytechnical University, Xi’an 710072, China; wyp721@nwpu.edu.cn (Y.W.); hbw123@live.cn (B.H.); mxgie@mail.nwpu.edu.cn (X.M.); ychwang@nwpu.edu.cn (Y.W.); weili2019@nwpu.edu.cn (W.L.); 2 Research & Development Institute of Northwestern Polytechnical University, Shenzhen 518000, China; 3 Yangtze River Delta Research Institute of NPU, Taicang 215400, China

**Keywords:** heterogeneous immunoassay, induced charged electroosmosis, sinusoidal bipolar electrode, microfluidic chip

## Abstract

In the context of the COVID-19 epidemic, enhancing the transport of analyte to a sensor surface is crucial for rapid detection of biomolecules since common conditions, including low diffusion coefficients, cause inordinately long detection times. Integrated microfluidic immunoassay chips are receiving increasing attention for their low sample volume and fast response time. We herein take advantage of asymmetric ICEO flow at a bipolar sinusoidal electrode to improve the rate of antibody binding to the reaction surface based on finite element modeling. Three different microfluidic cavities are proposed by changing the positions of the surface reaction area. We further investigate the relationship between binding enhancement and reaction surface positions, Damkohler number, and the voltage and frequency of the AC signal applied to the driving electrodes. Furthermore, the influence of the AC signal applied to the sinusoidal bipolar electrode on antigen–antibody-binding performance is studied in detail. Above all, the simulation results demonstrate that the microfluidic immune-sensor with a sinusoidal bipolar electrode could not only significantly improve the heterogeneous immunoassays but also enable efficient enhancement of assays in a selected reaction region within the micro-cavity, providing a promising approach to a variety of immunoassay applications, such as medical diagnostics and environmental and food monitoring.

## 1. Introduction

SARS-CoV-2 has caused the COVID-19 pandemic with a significant global impact. Coronaviruses cause mild to moderate upper respiratory tract illnesses in both humans and animals [1]. To contain the outbreak and manage the pandemic, early diagnosis and screening of COVID-19 patients are necessary since rapid isolation of COVID-19 patients prevents spreading events and facilitates the timely treatment at the early state of the illness. Real-time reverse transcription-polymerase chain reaction (RT-PCR) has been approved by the US Food and Drug Administration (FDA) to detect SARS-CoV-2. However, molecular diagnosis based on RT-PCR usually requires expensive reagents, skilled staff, and specific equipment. Besides, the preparation step is time-consuming, complicated, and easily influences diagnostic accuracy [2]. Thus, molecular diagnostic tests are not suited for point of care testing for COVID-19 or other diseases.

By developing specific immunoassays to detect antigen proteins, the SARS-CoV-2 antigen can be directly detected based on immunological diagnostic tests, which are reliable, cheap, timely, and widely used for the diagnosis of acute infection. Immunoassays, depending on specific antigen–antibody binding reactions, have attracted enormous attention in various fields, such as medical diagnostics and environmental and food monitoring [3]. Conventional immunoassays involve complex detection protocols and require qualified professionals, limiting the wide range of applications due to inherent diffusion-limited reaction kinetics, and an incubation step of hours or even longer for achieving a detectable level [4]. With no signal amplification technique for proteins and peptides, limit of detection (LOD) can merely be decreased by sacrificing detection sensitivity. Recently, new formats for miniaturized immunoassays have gained extensive attention. Microfluidic technology [5], a nascent technology, seeks to alleviate the above dilemmas, and the performance of analysis can be upgraded by requiring small amounts of reagents, reducing the analysis time, improving the sensitivity and reliability through automation, and integrating multiple processes in a simple chip. Immunoassays are ported onto microfluidic formats increasingly due to enormous challenges and unmet medical needs such as early diagnosis of disease by detecting ultra-low concentration analytes, clinical trials of new drugs, and the rapid detection of disease in resource poor areas [6]. The combination of microarray technologies and microfluidic chips provides the capability to simultaneously detect many different samples in a small area, which is quite useful in proteomics (protein microarrays) and genomics (DNA microarrays). However, they are still limited, arising from diffusion limitations of the analyte in the laminar flow regime, where large analyte depletion layers act as a resistance to analyte detection, and a long time is required for delivering an analyte to the biosensor at lower concentrations [7]. To address this problem and further reduce LOD, analytes can be transported to the sensor by active delivery with an external force, such as electric [4], magnetic, or acoustic force. Some scholars have utilized pumping techniques to improve the binding performance. A passive mixing structure is placed over the capture spot by Lynn et al. [7], to improve the binding rate of analyte to the sensing surface.

Many passive mixers have been used to improve the binding performance and enhance the signal generation, but more samples are required, and the geometry is much more complex [8,9]. Although the method of flow confinement [10] can confine the sample into a thin layer above the sensor surface, the depletion layer is not eliminated completely. AC electrokinetics, including dielectrophoresis (DEP), AC electrothermal (ACET) and AC electroosmosis (ACEO), have been widely used to enhance the performance of heterogeneous immunoassays thanks to the advantages of requiring low AC power and alleviating electrolysis reactions [3,11]. DEP [12,13] is often used to manipulate particles based on the difference in polarizability between a particle and the medium. Cheng et al. use DEP to trap the concentrated molecules at the surface of an antibody-immobilized electrode. Li et al. [14,15] presents a sensitive affinity sensor by integrating DEP into label free capacitive measurements. Nevertheless, DEP is size-dependent and short-ranged, limiting the detection and manipulation of small biomolecules [16]. ACET is caused by the movement of induced charges due to the joule heating in the fluid under the influence of electric fields [17]. Many scholars [3,18,19] have demonstrated the capability for ACET effect [17] to improve the detection of diluted and small molecule targets. Sigurdson et al. [3,19] demonstrate the ability of ACET flow to enhance the performance of heterogeneous assays. Selmi et al. [20] numerically study the effect of the electrothermal flow on the binding reaction of C-reactive protein (CRP). Though ACET flow can transport small molecules to the sensor surface and enhance binding assays, it usually causes large temperature rise. ACEO flow, arising from the interaction of the electric field with the charge in the diffuse double layer [21], can easily generate large velocities using low voltages and has also been investigated to enhance the binding performance [22]. Wu et al. integrate ACEO flow and label-free electrochemical impedance spectroscopy in DNA sensing chips [23]. The reaction surface is usually located at the driving electrode surface due the circulating ACEO flow near the electrode, which is not desirable for analyzing immunoassays in multiple or different reaction regions. Recently, based on the optically induced ACEO flow, Han et al. [24,25] reported a novel optoelectrofluidic immunoreaction system for enhancing antibody analyte binding performance, permitting the detection of target analyte in low sample volumes. The microarray-integrated optoelectrofluidic immunoassay system can not only result in the consumption of small amounts of both precious samples and expensive antibodies but also enable the assays to be run in parallel efficiently [26]. However, the optoelectrofluidic system requires complex fabrication processes (the preparation of the photoconductive substrate) and illumination components, limiting its practical application in diverse fields [27]. Merkoci et al. [28] explores micromotors-assisted microarray technology for immunoassays; however, the use of hydrogen peroxide might degrade the involved proteins, and the accumulation of bubbles occurs in the chip.

We herein take advantage of asymmetric induced charged EO (ICEO) flow at a sinusoidal bipolar electrode (BPE) to improve the rate of antibody binding to the reaction surface based on finite element modeling. Ren et al. [29] exploited ICEO flow in the rotating field for enhancing immunoassays, but the use of a rotating electric field limits its wide application, and it is difficult to enhance the binding reactions in a specific region. In contrast, the introduction of fixed-potential ICEO flow at a sinusoidal BPE in this work can permit the enhancement of assays in a selected reaction region and enable efficient immunoassays in an array wirelessly. This work aims to understand how the positions of the reaction region affect assay improvement and which optimum frequency and voltage to use, and to investigate the influence of the gate voltage of the sinusoidal electrode on antibody binding performance. Asymmetric induced charge electroosmosis (ICEO) flow is utilized to stir the flow field above the BPE electrodes, accelerate the transport of analyte to the functionalized surface, and simultaneously minimize the localized target depletion. An optimized design of the proposed microfluidic chip is proposed based on the immunoassay enhancement. As a result, the sensor target interaction can be improved, and it owns the capability of the efficient enhancement of assays in a selected reaction region within the microchip. The current asymmetric-ICEO-flow-assisted microarray technology can also be extended to other proteins, DNA, and cell analyses.

## 2. Materials and Methods

### 2.1. Theoretical Background

#### 2.1.1. Induced Charged Electroosmosis

Traditional electroosmotic flows occur [30] when an applied electric field forces the thin ionic clouds, which screen charge surfaces into motion. Unlike the former, induced charge electroosmosis (ICEO) phenomena [31,32,33,34] arise when the diffuse double layer charge is induced around polarizable surfaces by an applied electric field and subsequently the same electric field drives the induced charge into motion [35]. So, the fundamental difference is the origin of the diffuse double layer charge. In ICEO, the double layers are provided by electrical conductors that may not be energized, whereas in ACEO, the double layers are formed on the electrode surfaces [36].

The typical model of ICEO contains the Poisson–Nernst–Planck equations of ion transport coupled to the Navier–Stokes equations of the viscous fluid flow. Based on the assumption of weakly nonlinear or linear charging dynamics, we can simplify this standard ICEO model by decoupling the electrokinetic issue into viscous flow and electrochemical relaxation [37].

The electric potential in the solution can be obtained in terms of Laplace’s equation:

(1) $$\nabla \cdot \left(\sigma E\right)\;=-\sigma \nabla^{2}\phi =0$$

assuming electrolytes with a constant conductivity σ.

A compact Stern layer is often assumed to act as a capacitor in series with diffuse layer capacitor; the total induced double layer (IDL) capacitance is $C_{0}=C_{S}C_{D}/\left(C_{S}+C_{D}\right)\;=C_{D}/\left(1+\delta \right)$, and the voltage across the diffuse layer capacitor only occupies a portion of the total double layer voltage $\psi_{D}=\Delta \phi /\left(1+\delta \right)$, where $C_{D}=\varepsilon /\lambda_{D}$ is the capacitance of the diffuse layer and $C_{S}$ is the capacitance of the Stern layer. $\delta =C_{D}/C_{S}$ is the ratio of the diffuse layer to Stern layer capacitance, ε is the permittivity, and $\lambda_{D}=\sqrt{D\varepsilon /\sigma }=37.6$ nm is the Debye screening length, where $D=2\times 10^{-9}m^{2}/s$ is the bulk diffusivity and $\varepsilon =7.08\times 10^{-10}F/m$ is the permittivity.

A capacitance-like boundary condition closes the equivalent RC circuit and the normal current from the bulk charges the diffuse layer:

(2) $$C_{0}\frac{d\psi_{0}}{dt}=-\sigma \hat{n}\cdot \nabla \phi =\sigma E_{n}$$

Using complex amplitudes, the above condition can be written as

(3) $$jwC_{0}\frac{\tilde{\phi }-{\tilde{\phi }}_{0}}{1+\delta }=\sigma \hat{n}\cdot \nabla \tilde{\phi }$$

where $\tilde{\phi }$ is the potential in the bulk outside the IDL and ${\tilde{\phi }}_{0}$ is the potential at the metal surface.

The boundary condition at the insulating surface can be given by

(4) $$\frac{\partial \phi }{\partial y}=0$$

Based on the Helmholtz–Smoluchowski boundary condition, the time-averaged flow for the slip velocity on the polarizable surface is obtained as follows:

(5) vs =−ε2ηReζ˜Et˜* =−εη11+δ12Reϕ0˜−ϕ˜E˜−E˜⋅n⋅n*

where $E_{t}$ denotes the tangential electric field, ζ denotes the zeta potential, η denotes the viscosity of fluid sample, p denotes the pressure, and the asterisk indicates complex conjugation.

The slip-free boundary condition is applied to other surfaces, including the insulating surface and driving electrode surface.

(6) v=0

**Figure 1:**
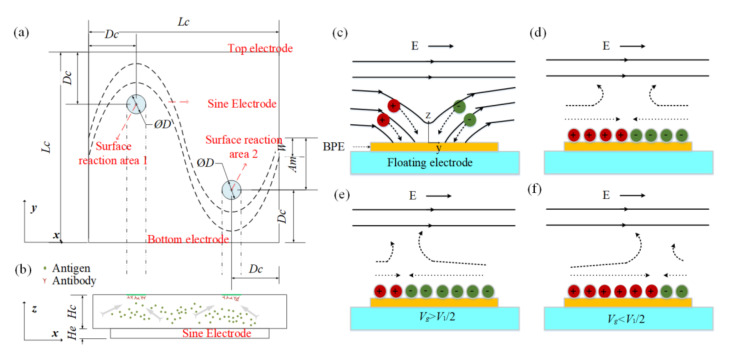
(**a**) Top view and (**b**) side view of the proposed chip for enhancing the heterogeneous immunoassay in a static microchamber. Suspended antigens in the bulk are transported toward the reaction surface based on the ICEO flow above a sinusoidal bipolar electrode. The illustration of the basic physics behind ICEO: (**c**) as soon as the electric field is applied, the electric field lines perpendicularly intersect the electrode at first, driving ions from solution onto the sinusoidal electrode. (**d**) When the double layer is formed fully, the electric field is screened from the sinusoidal electrode and parallel to the electrode surface, resulting in a slip velocity and two counter-rotating rolls by driving the ions in the double layer. (**e**) When the sinusoidal electrode is energized with *V*_g_ (*V*_g_ > *V*_1_/2), a negatively charged double layer forms, which drives the fluid away from the grounded electrode, giving rise to asymmetric vortexes. (**f**) A positively charged double layer occurs when the sinusoidal electrode is energized with *V*_g_ (*V*_g_ < *V*_1_/2), driving the fluid towards the grounded electrode.

The evolution of an induced double layer is shown in Figure 1c,d. In view of the equipotential nature of the electrode surface, the electric field lines intersect the central electrode surface at right angles as soon as an electric field is applied, then ions in solution are driven along field lines and gradually accumulate on the electrode surface to form a double layer. There is no net charge in the induced double layer. The positive ions and negative ions are deposited close to the field source and field sink, respectively. When the applied electric frequency is low, the induced double layer (IDL) will reach steady state and all field lines will be expelled from the electrode; thus, the conducting surface can be treated as an insulator. Then, the tangential electric field drives the induced charge into motion and generates micro-vortices around the electrode surface, resulting in a flow stagnation region at the center of the floating electrodes shown in Figure 1c,d. However, most of the applied voltage will be dropped across the bulk if the frequency is high, and the central electrode will recover to a perfect conductor. 

As for fixed-potential ICEO, an analytical solution for the induced zeta potential in the DC limit can be achieved as follows,

(1) When the gate electrode is floating, the induced zeta potential is
  (7) $$\zeta \left(t\right)\;=\frac{1}{1+\delta }\left(\frac{V_{1}}{2}\cos(wt)-\phi \left(t\right)\right)\;=\frac{1}{1+\delta }Ex\cos(wt)$$

The ICEO slip velocity on the surface of the floating electrode is given by the Helmholtz–Smoluchowski equation:

(8) $$\langle v_{s}\rangle \;=\frac{-\varepsilon }{\eta }\langle \frac{1}{1+\delta }Ex\cos(wt)\cdot E_{t}\rangle =\frac{-\varepsilon E^{2}x}{2\eta \left(1+\delta \right)}$$

(2) Assuming the phase gap $\theta_{g}$ = 0, when an electric signal $V_{g}\cos(wt)$ is applied to the gate electrode, the zeta potential becomes:
  (9) $$\zeta \left(t\right)\;=\frac{1}{1+\delta }\left(V_{g}\cos(wt)-\phi \left(t\right)\right)\;=\frac{1}{1+\delta }\left(Ex\cos(wt)+\left(V_{g}-\frac{V_{1}}{2}\right)\cos(wt)\right)$$

The ICEO slip expression becomes:

(10) $$\langle v_{s}\rangle \;=\frac{-\varepsilon }{\eta }\langle \frac{1}{1+\delta }\left(Ex\cos(wt)+\left(V_{g}-\frac{V_{1}}{2}\right)\cos(wt)\right)\cdot E_{t}\rangle \;=\frac{-\varepsilon E}{2\eta \left(1+\delta \right)}\left(Ex+V_{g}-\frac{V_{1}}{2}\right)$$

If $\langle v_{s}\rangle$ is zero, the flow stagnation line (FSL) can be easily obtained; the stagnation line is located at the BPE center (*x* = 0) when no signal is applied at the gate electrode. The FSL can be changed ($x=\frac{1}{E}\left(\frac{V_{1}}{2}-V_{g}\right)$) by changing the electric signal $V_{g}\cos(wt)$. For example, when the gate electrode is driven by $V_{g}\cos(wt)$($V_{g}>V_{1}/2$), positive charges will be accumulated at the BPE surface, causing the generation of a negatively charged double layer in the solution near the BPE surface. Therefore, an asymmetric ICEO flow forms and FSL is propelled away from the grounded electrode, as shown in Figure 1e. Besides, when $V_{g}<V_{1}/2$, a positively charged double layer occurs and FSL is transported toward the grounded electrode (Figure 1f). Through varying the electric potential on the BPE, the zeta potential above the electrode can be adjusted, contributing to shifting the flow stagnation line and producing asymmetric flow across the channel. The direction of the asymmetry can also be altered by varying the potential of the gate electrode.

#### 2.1.2. Immunoassay Surface Reaction

Immunoassays are ubiquitous and depend on the excellent specificity of antigen–antibody reactions to detect target proteins such as viruses and bacteria. Heterogeneous immunoassays include a surface-based attachment between an antigen and an antibody. Antibodies can be first immobilized on the reaction region while antigen is contained in the sample to react with the surface. 

The analyte solution is injected into the microchamber with a concentration of *C*_0_. The distribution of the antigen in bulk sample is determined based on the traditional convection-diffusion equation, as follows:

(11) $$\frac{\partial C}{\partial t}+\vec{u}\cdot \nabla C=D\nabla^{2}C$$

where *C* is the concentration of the antigen in the bulk solution, *D* is the diffusion coefficient of the antigen, and $\vec{u}$ is the flow velocity.

The antibody is immobilized at the bottom surface of the microchannel first. The biding reaction in a surface binding event can be described as follows:

(12) $$C+B\overset{k_{ads}}{\underset{k_{des}}{⇄}}C_{s}$$

where *B* is the concentration of binding sites at the reaction surface and *C_s_* is the concentration of the antigen–antibody complexes at the surface. *k_ads_* is the association rate constant, and *k_des_* is the dissociation rate constant, respectively.

The rate of change of the bound species at the reaction region, including surface diffusion and the reaction for its formation, is described as follows:

(13) $$\frac{\partial C_{s}}{\partial t}=D_{s}\nabla^{2}C_{s}+k_{ads}CB-k_{des}C_{s}$$

where *D_s_* is the diffusion coefficient of the bound species at the reaction surface.

An analytical expression for the surface concentration of the bound complexes at equilibrium can be achieved by ignoring the spatial diffusion term $D_{s}\nabla^{2}C_{s}$,

(14) $$C_{s}^{eq}=\frac{k_{ads}B_{0}C}{k_{ads}C+k_{ads}}$$

where *B*_0_ is the receptor concentration at the reaction surface. The concentration of the available sites, *B*, is the difference between the number of the occupied sites *C_s_* and the initial concentration of sites *B*_0_. The rate of binding at the surface for a first order reaction is *k_ads_CB* = *k_ads_C*(*B*_0_ − *C_s_*), and the rate of dissociation is *k_des_ C_s_.* The time rate of variation of antigen bound to the immobilized antibodies is shown as follows:

(15) $$\frac{\partial C_{s}}{\partial t}=D_{s}\nabla^{2}C_{s}+k_{ads}C\left(B_{0}-C_{s}\right)-k_{des}C_{s}$$

The rate of antigen binding to immobilized antibodies should be balanced by the diffusive flux of analyte at the reaction surface. The boundary condition at the binding surface is expressed based on mass flux:

(16) $$\frac{\partial C_{s}}{\partial t}=D\frac{\partial C}{\partial y}\left|\underset{y=0}{}\right.$$

A binding enhancement factor, be = *C_s_*_1_/*C_s_*_0_, is used to quantify the performance of asymmetric ICEO flow vortexes in enhancing the antigen–antibody binding rate. *C_s_*_1_ and *C_s_*_0_ indicate the concentration of the bound complexes at the surface with and without applying an AC signal.

As the reaction transport species from the sample near the surface, concentration gradient will appear and interact with fluid flow, making the calculation of *C* nontrivial, which is used to concurrently solve the flow fields and the mass transport in the bulk solution.

Based on Damkohler (*Da*) number, we can measure whether a reaction is diffusion-limited or reaction-rate-limited. *Da* is a ratio of reaction velocity (*k_ads_ B*_0_) to the rate of transport (*D*/*h*). *Da* > 1 means a transport-limited system, while *Da* < 1 indicates a reaction-rate-limited system. When the analyte is a large molecule, *Da* will be large because of its small diffusion coefficient.

### 2.2. Methods

#### 2.2.1. Device Design

Three microfluidic chips are shown in Figure S1. A sinusoidal bipolar electrode is designed on the bottom of the square microchannel. An AC signal is applied to the top and bottom driving electrodes. The only difference between these three chips is the position of surface reaction areas at the top of the microchannel. The structure parameters of the optimized chip are shown in Figure 1a, and Table 1 and Table 2 shows the position parameters of the surface reaction areas in these three structures.

#### 2.2.2. Numerical Solver

A commercial software package (COMSOL Multiphysics 5.3a) is used to simulate the kinetics reaction of the proposed microfluidic immunoassay. The electric field distribution, flow pattern, transportation of antigen, and surface reaction under different conditions are simulated in detail.

## 3. Results and Discussion

To evaluate the effect of some critical parameters on the kinetics of the microfluidic immunoassays, we conduct some simulations using the proposed designs. The parameters for SARS-CoV-2 antigen and antibody used in this work are shown as follows: an immobilized antibody concentration *B* = 3.3 × 10^−8^ mol/m^2^, the association rate constant *k_ads_* = 197 m^3^/(s·mol), the dissociation rate constant *k_des_* = 2.58 × 10^−4^ (1/s), the concentration of the SARS-CoV-2 antigen in the bulk *C*_0_ = 1 × 10^−7^ mol/m^3^, and the diffusion coefficient of the antigen *D* = 2 × 10^−12^ m^2^ s^−1^, which are comparable to the values reported in other studies [38]. We analyze the grid independence in Supporting Information. As shown in Figure S2, surface slip velocity versus different grid elements is investigated. When element numbers are 35,121 and 112,223, the surface slip velocities are 1.5103 mm/s and 1.5302 mm/s, respectively, and the standard deviation is 1.4074 × 10^−5^. Above all, we can consider that the grid division method is independent after element number exceed 35,121. In this work, we divide 35,121 meshes for the following numerical simulations.

### 3.1. Binding Enhancement by a Sinusoidal Bipolar Electrode

**Figure 2:**
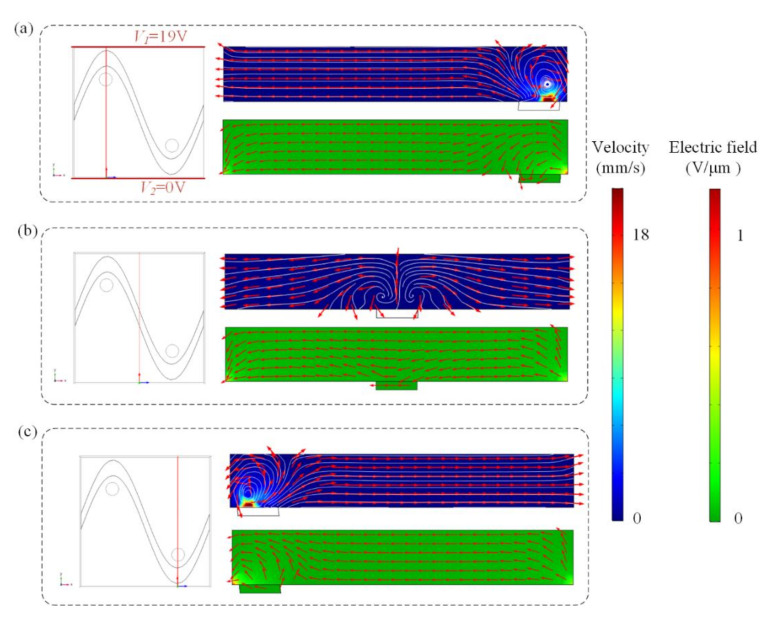
Flow velocity and electric field distribution on the bipolar sinusoidal electrode at different cross-sections. The cross-section is at (**a**) x = 250 μm, (**b**) x = 125 μm, and (**c**) x = 375 μm. Only two lateral driving electrodes (red lines in a) are applied with AC signals of *V*_1_ = 19 V, *V*_2_ = 0 V, and *f* = 80 Hz, respectively. Top row: fluid flow vector in the transverse section of the channel. Bottom row: electric field distribution in the transverse section of the channel at (**a**) x = 250 μm, (**b**) x = 125 μm, and (**c**) x = 375 μm.

Swirling ICEO flow pattern can be used to circulate suspended antigen past the binding surface, offering more binding opportunities for the suspended molecules. Flow velocity and electric field distribution at different cross-sections predicted by the numerical model are shown in Figure 2, when the top and bottom electrodes are energized with AC signals of *V*_1_ = 19 V, *V*_2_ = 0 V, and *F* *=* 80 Hz and the sinusoidal bipolar electrode is floating. Figure 2a,c indicate that a higher AC field strength and stronger asymmetric ICEO flow vortexes occur near the region where the driving electrode is located nearest to the sinusoidal electrode. Symmetric ICEO flow vortexes also appear near the middle region of the microcavity in Figure 2b. Because the high flow velocity can be generated near the region where the driving electrode is close to the sinusoidal electrode, we at first expect the opposite surface of the region to be an optimum sensor location. The sinusoidal electrode offers asymmetric vortexes for enhancing antigen–antibody binding by micro-stirring.

**Figure 3:**
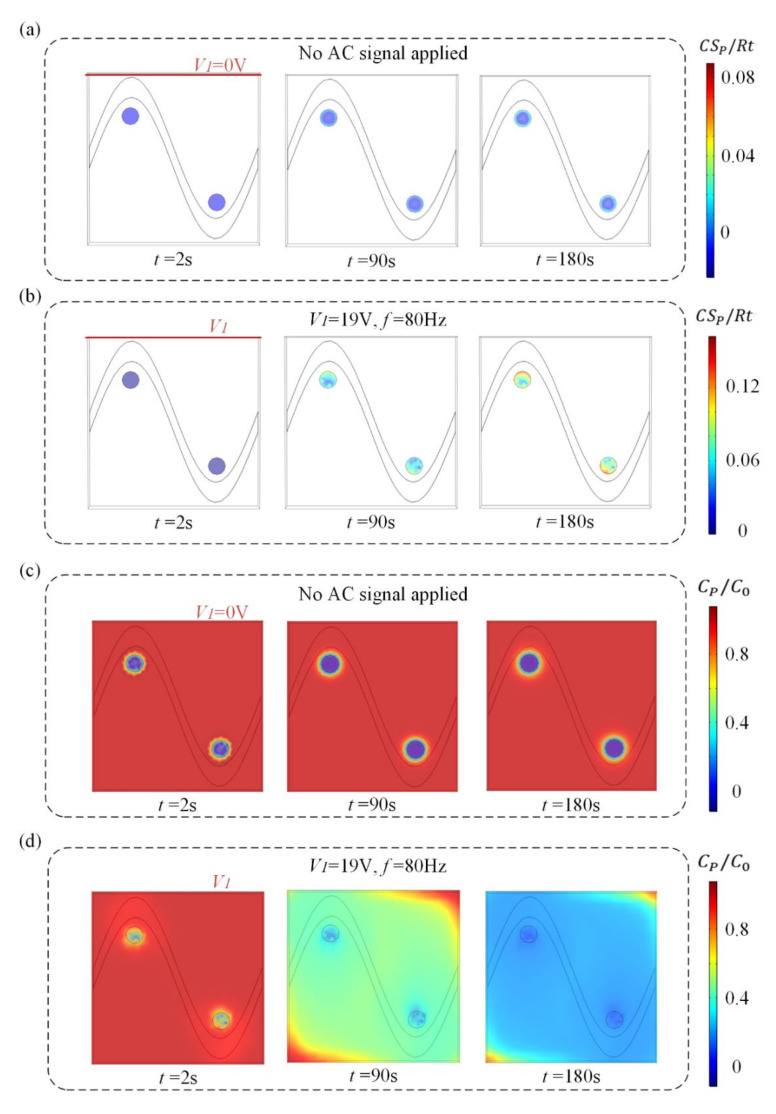
(**a**,**b**) Normalized distribution of surface concentration field of bound antigen–antibody complexes and (**c**,**d**) bulk concentration field of antigen over time for the following cases. When no electric field is applied, (**a**) surface antigen–antibody complexes distribution and (**c**) bulk antigen distribution over time indicate that the suspended antigen concentration is depleted merely by diffusion in the absence of ICEO micro-stirring. After the driving electrodes are energized with an AC signal of *V_1_* = 19 V and *f* = 80 Hz, the ICEO vortexes redistributed (**b**) the surface antigen–antibody complexes distribution and (**d**) bulk antigen distribution.

We investigate the effect of this flow pattern on the binding response of a heterogeneous immunoassay in which antibody has been immobilized at the surface reaction region. Simulation results in Figure 3 show the surface concentration field of bound antigen–antibody complexes and suspended antigen concentration field in the micro-cavity over time. In a standard and passive condition where the chip is not energized with an AC signal (Figure 3a,c), diffusion is the only transport mechanism and binding process depletes the suspended analyte concentration, ensuring that the depleted region near the reaction surface grows with time and decreases the binding rate of the immunoassay. Figure 3a,c show the surface concentration distribution of bound antigen–antibody complexes and suspended antigen concentration distribution before applying an AC signal, indicating that the immunoassay is implemented in the mass-transport-limited regime and the majority of the surface binding of antigen occurs near the reaction region. As the antigen binds to the reaction surface, a “depletion zone” quickly appears above the binding surface. Thus, a decreasing in the assay time requires the overcoming of the sharp reduction in the antigen binding near the reaction surface.

Figure 3b,d display the surface concentration field of bound antigen–antibody complexes and suspended antigen concentration field over time after applying an AC signal of *V*_1_ = 19 V, *V*_2_ = 0 V and *f* = 80 Hz. The reason for the apparent change in bound antigen–antibody complexes is mainly due to the accelerated transmission of antigen to the surface reaction areas after applying an electric field. With the application of an electric field, ICEO vortexes are generated at the bipolar electrode surface and the micro-stirring over the BPE surface accelerates the fluid flow over the reaction surface, redistributing the depleted antigen concentration (Figure 3d). The near-wall deleted concentration is replenished with fresh antigen, thus promoting a uniform and rapid distribution of antigen concentration throughout the chamber. Figure 3b shows a greater amount of antigen–antibody complexes bound at the reaction surface, which can be attributed to efficient transport of suspended antigens to the reaction surface.

### 3.2. Effect of the Position of the Reaction Surface, Damkohler Number, Applied Voltage, and Frequency

**Figure 4:**
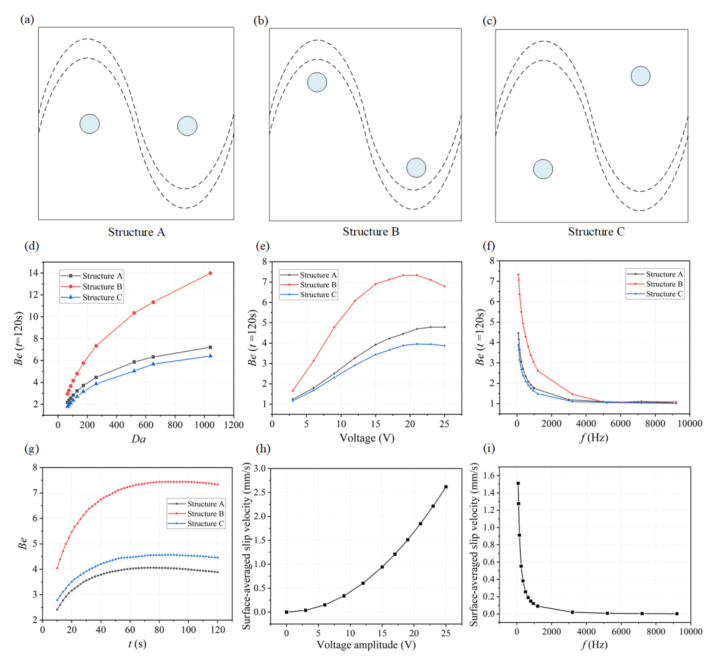
The influence of the positions of reaction surface, Da number, the applied voltage, and frequency on the binding enhancement factor Be. (**a**,**c**) Microfluidic devices designed with three different structures ((**a**) Structure A, (**b**) Structure B, and (**c**) Structure C) by changing the positions of binding surface. (**d**) Simulation results showing that the binding enhancement factor Be increases with increasing Da number when applying a signal of *V*_1_ = 19 V and *f* = 80 Hz for three different designs. Each point indicates two time-dependent simulations: one with and one without ICEO flow stirring; reported value is the ratio of the antigen–antibody complexes at 120 s for these two simulations. (**e**) Be with respect to AC signals with different voltage amplitudes applied at t = 120 s when Da = 260.04. (**f**) Be as a function of applied frequency when applying a signal of *V*_1_ = 19 V and *f* = 80 Hz for Structure B at t = 120 s, Da = 260.04. (**g**) Plot of Be versus assay time under an AC field of *V* = 19 V and *f* = 80 Hz. (**h**) Numerical results of the surface-averaged slip velocity for different voltage amplitudes when Da = 260.04 and *f* = 80 Hz. (**i**) A plot of surface-averaged slip velocity versus the applied frequencies at lateral electrodes when Da is 260.04 and *V*_1_ = 19 V.

As the ICEO flow driven circulation redistributes the depleted concentration throughput the micro-cavity, the position of the functionalized reaction surface needs to be investigated for achieving a higher binding rate. As illustrated in Figure 4c, we designed three microfluidic chips with different locations of reaction surface regions. The coordinates of the central position of circular reaction surfaces are given in Table 2. Simulations have been carried out for these three structures under different conditions.

To demonstrate the effectiveness of ICEO flow stirring above sinusoidal BPE surface in a variety of applications, we first study the influence of the Damkohler number (*Da*) upon binding rate. As for large *Da*, slow diffusion limits the binding rate, and any improvement in transport of analyte to the reaction surface or through ICEO-flow driven micro-stirring will enhance the binding performance. A series of numerical simulations are carried out for different *Da*. The binding enhancement factor *Be*= *C_s_*_1_/*C_s_*_0_ is defined as the ratio of bound antigen–antibody complexes from ICEO micro-stirring to the bound complexes without micro-stirring.

For each parameter set, the binding enhancement factor *Be* is calculated at *t* = 120 s (Figure 4d,f). According to the parameters of SARS-CoV-2 antigen and antibody, here we choose *D* = 3 × 10^−12^ m^2^ s^−1^ as the diffusion coefficient for the following simulation analysis (*Da* = 260.04). We also further studied the binding performance when *Da* is larger than 500 by changing the diffusion coefficients as shown in Figure 4d. It is noted that the effect of ICEO flow for enhancing immunoassays using these three structures is becoming more significant. As shown in Figure 4d, the antigen–antibody binding efficiency increases with increasing *Da* number, especially for Structure B. According to this plot, we can predict how much binding enhancement can be generated by ICEO flow vortexes at a sinusoidal electrode. The ICEO flow micro-stirring in Structure B yields an enhancement factor of 10 higher binding for *Da* = 500. The results show that for all values of *Da*, a reasonably significant enhancement factor can be obtained in Structure B compared to other structures including Structure A and C. 

The applied voltage and frequency will both influence the fluid velocity and binding performance. To find the appropriate voltage, simulations to determine the suitable voltage are conducted by varying voltage ranging from 2.5 V to 25 V based on the Structure B. The binding enhancement factor *Be*, as a function of the applied voltage in the three structures when *Da* is 260.04 at 120 s, is shown in Figure 4e, illustrating *Be* generally increases with increased applied voltage, and an extreme value of *Be* (about 7.5) occurs when the applied voltage is 20 V in Structure B. When the applied voltage is small, the transportation of antigen to the reaction surface is accelerated as the voltage magnitude increases, thus enhancing the binding performance. However, when the voltage amplitude is too high, the value of *Be* increases slowly or even decreases because the reaction rate and the ICEO flow induced convection rate cannot be matched. The antigen–antibody binding reaction regime changes from being transport-limited to being reaction-limited due to the high ICEO flow velocity at high voltages. To obtain the ideal operating conditions for the immunoassays, an optimum frequency is investigated by varying frequency ranging from 1 Hz to 9 kHz based on these three structures. The dependence of the *Be* on the applied frequency is shown in Figure 4f for these three microfluidic chips, and *Be* at *t* = 120 s decreases with increasing frequency because the applied potential drops completely across the electrolyte and the induced surface charges in double layer tend to zero at higher frequencies, leading to weaker ICEO vortexes. The lowest frequency used is 80 Hz, to avoid hydrolysis and electrode damage. From the comparison of *Be* for the three structures in Figure 4d,g, we choose structure B for the subsequent simulation analysis. The predicted transient process of the antigen–antibody binding at the reaction region using three different designs is depicted in Figure 4g, indicating the average improvement in antigen–antibody binding during different assay durations. The improvement factors are 7.2, 4.5, and 4 for one minute of ICEO flow stirring in Structure B, Structure C, and Structure A, respectively, and increase steadily for longer assay times. After about 80 s, the increases in binding efficiency approach a maximum value and remain fairly constant for these three structures, demonstrating the effectiveness of ICEO flow stirring above a sinusoidal bipolar electrode to enhance binding rates. Figure 4h,i show the effect of applied AC signal frequency and amplitude on surface-averaged slip velocity at *Da* = 260.04 for the Structure B. The binding efficiency versus the inlet flow rate when *Da* = 260.04 and *V*_1_ = 19 V, *f* = 80 Hz at *t* = 120 s can also be found in Figure S3. When the flow velocity is under 50μm/s, the binding efficiency improves significantly because the interaction of the ICEO flow and the inlet pressure flow facilitates the transport of antigen to the surface reaction areas. Note that when a higher flow rate of the inlet pressure flow (larger than 100 μm/s) is introduced, the pressure flow dominates over the ICEO flow, which decreases the binding enhancement factor of ICEO flow.

### 3.3. Effect of Gate Voltage at the Sinusoidal Bipolar Electrode

**Figure 5:**
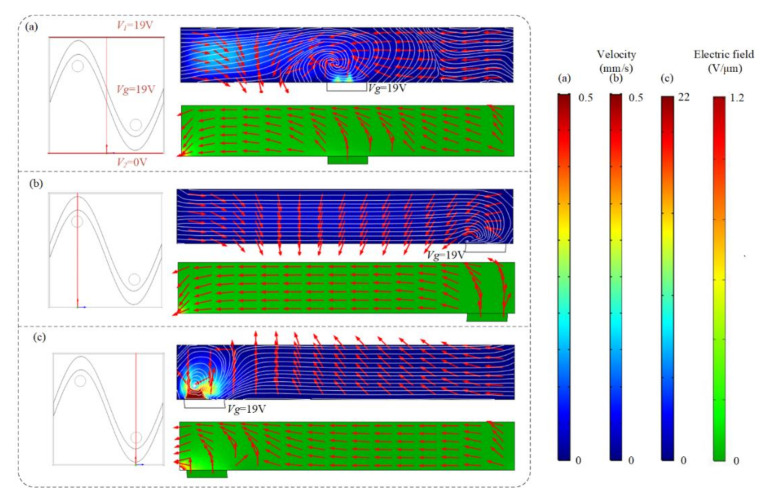
The asymmetrical distribution of the flow field and electric field at different cross-sections ((**a**) x = 250 μm, (**b**) x = 125 μm, and (**c**) x = 375 μm) after applying an AC signal with a voltage of 19 V and a frequency of 80 Hz to the sinusoidal electrode.

We previously have demonstrated the influence of ICEO flow above the sinusoidal floating electrode on the binding enhancement. Here, we also investigate how the AC signal exerted on the sinusoidal bipolar electrode will influence the binding performance when the driving electrodes are energized with AC potentials of *V*_1_ = 19 V at *f* = 80 Hz. Figure 5 shows the asymmetrical distribution of vortex flow and electric field within the microchannel at different cross-sections when the driving electrodes and sinusoidal bipolar electrode are driven with AC signals of *V*_1_ = 19 V, *V*_2_ = 0 V, *V*_g_ = 19 V, and *f* = 80 Hz. Figure 5a illustrates that the cross-sections of the flow field and electric field distributions at x = 250 μm, displaying that a high electric field and strong asymmetric ICEO flow vortexes form near the region where the sinusoidal electrode located nearest the ground electrode at *V*_1_ = 19 V, *V*_2_ = 0 V, *V*_g_ = 19 V, and *f* = 80 Hz. In Figure 5b, a large distance between the sinusoidal electrode and ground electrode exists and results in a lower electric field and weaker ICEO flow velocity field. In contrast, a much higher ICEO flow field and electric field can be generated near the region where the ground electrode is close to the sinusoidal electrode in Figure 5c, contributing to the enhancement of antigen–antibody binding by micro-stirring.

**Figure 6:**
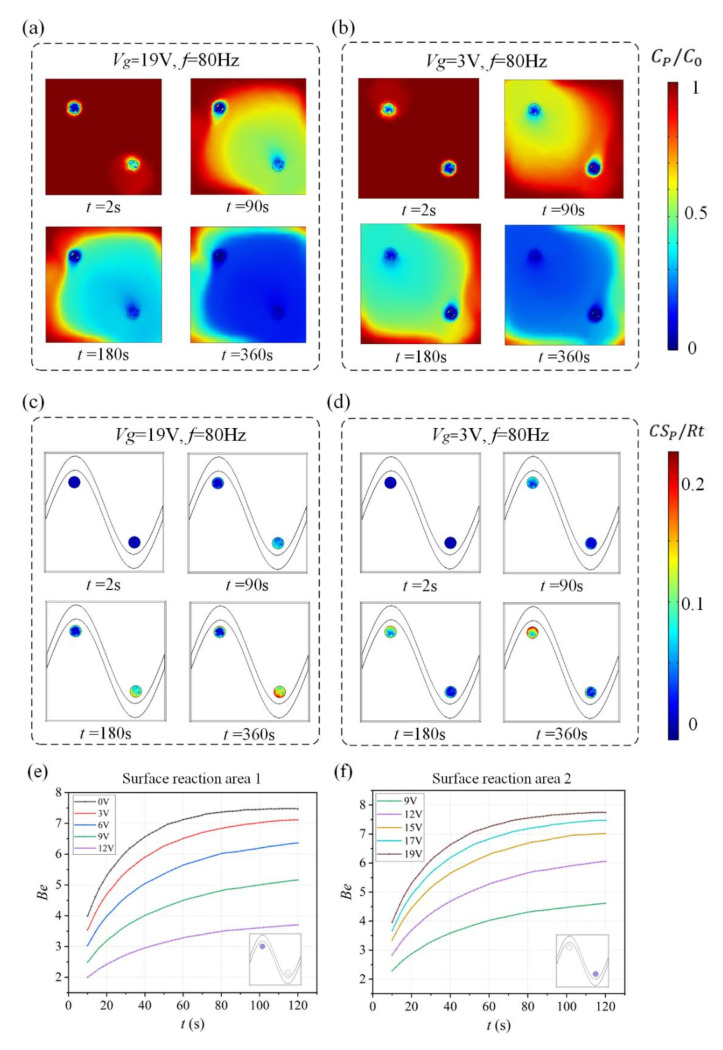
(**a**,**b**) Normalized distribution of bulk concentration field of antigen and (**c**,**d**) surface concentration field of bound antigen–antibody complexes as well as the enhancement factor Be over time for Structure B when the sinusoidal bipolar electrode is driven with different gate voltages. (**a**) Bulk antigen distribution and (**c**) surface antigen–antibody complexes distribution over time when AC signals of *V*_1_ = 19 V, *V*_g_ = 19 V, and *f* = 80 Hz are applied to the driving and sinusoidal electrodes. After the driving electrodes are energized with AC signals of *V*_1_ = 19 V, *V*_g_ = 3 V, and *f* = 80 Hz, the ICEO vortexes redistribute (**b**) the bulk antigen distribution and (**d**) surface antigen–antibody complexes distribution. (**e**) A plot of Be in surface reaction area 1(purple area) versus assay durations for different voltages applied to the sinusoidal electrode (*V*_g_ < *V*_1_/2). (**f**) A plot of Be in surface reaction area 2 (purple area) versus assay durations for different voltages applied to the sinusoidal electrode (*V*_g_ > *V*_1_/2).

With the ability to actively change the potential of the gate sinusoidal electrode, the asymmetric ICEO vortex can be used to flexibly generate effective mixing, further improving the binding rate of assays in a selected reaction region. The influence of the gate voltage of the sinusoidal bipolar electrode on the enhancement performance of the heterogeneous immunoassay is investigated in detail, as shown in Figure 6.

Simulation results in Figure 6a,c show suspended antigen concentration field and the surface concentration field of bound antigen–antibody complexes in the micro-cavity over time after applying an AC signal of *V*_g_ = 19 V (*V*_g_ > *V*_1_/2) to the sinusoidal electrode at *V*_1_ = 19 V, *V*_2_ = 0 V and *f* = 80 Hz. A large electric field forms and produces a strong asymmetric ICEO flow (Figure 5a) near the region where the sinusoidal electrode is located closest to the ground electrode at *V*_1_ = 19 V, *V*_2_ = 0 V, *V*_g_ = 19 V, and *f* = 80 Hz, facilitating a strong, continuous, and non-invasive mixing of the target molecule with the immobilized antibody in the reaction area 2. In this case, compared to the reaction area 1, strong ICEO vortexes generated at the bipolar electrode surface near the reaction area 2 redistribute the depleted antigen concentration, and the majority of the surface binding of antigen occurs near the reaction region 2. On the other hand, when the sinusoidal bipolar electrode is driven with an AC signal of *V*_g_ = 19 V (*V*_g_ < *V*_1_/2) at *V*_1_ = 19 V, *V*_2_ = 0 V, and *f* = 80 Hz, a strong asymmetric ICEO flow will appear near the region where the sinusoidal electrode is located furthest the ground electrode. As a result, antigen concentration distribution and the surface concentration distribution of bound antigen–antibody complexes within the cavity are shown in Figure 5b,d. The near-wall deleted concentration is replenished with fresh antigen in surface reaction area 1 due to strong ICEO flow micro-stirring, thus generating a uniform and rapid distribution of antigen concentration near surface reaction area 1. We finally explore the binding enhancement factor Be versus times at each surface reaction area by flexibly changing the voltages applied to the sinusoidal electrode, as depicted in Figure 6e,f. When *V*_g_ < *V*_1_/2, the binding enhancement can be improved in surface reaction area 1 with the decreasing voltage applied to the sinusoidal electrode. When the sinusoidal bipolar electrode is driven with an AC signal of *V*_g_ = 0 V (*V*_g_ < *V*_1_/2) at *V*_1_ = 19 V, *V*_2_ = 0 V, and *f* = 80 Hz, the binding enhancement factor Be in surface reaction area 1 can reach about 7.5 at 120 s. In contrast to reaction area 1, the depletion effect near surface reaction region 2 can be alleviated due to the strong ICEO micro-stirring as the sinusoidal electrode is driven by a gate voltage *V*_g_ (*V*_g_ > *V*_1_/2) and the binding performance is enhanced greatly with the increasing voltage exerted upon the sinusoidal electrode. 

Above all, the electric field distribution can be adjusted by energizing the sinusoidal bipolar electrode with different voltages, further enabling efficient and flexible enhancement of heterogeneous immunoassays in a specific reaction area and contributing to the reasonable selection of the location of binding surface. This proposed device using coplanar the sinusoidal BPE electrodes has several advantages, such as low cost, being easy to fabricate, better controllability, and high efficiency, since other methods usually require laser [39], micromotor [28], or complicated 3D structure [7,40].

## 4. Conclusions

This work demonstrates the feasibility of using ICEO flow above a sinusoidal electrode to reduce the detection time and enhance the sensitivity of immunoassays. By exploiting the ICEO micro-stirring effect on a sinusoidal electrode in three different structures, the greatest enhancement occurs by using Structure B since the ICEO whirlpools can produce more chances for the binding reaction through delivering the free antigens to the reaction surface. The dependences of binding enhancement factor on the applied voltage, frequency, and *Da* number are studied using simulation analysis. Besides, the effect of the AC signal applied to the sinusoidal bipolar electrode on the binding performance is investigated, and the binding enhancement factor *Be* in surface reaction area 1 can reach about 7.5 at 120 s when the sinusoidal bipolar electrode is energized with an AC signal of *V*_g_ = 0 V (*V*_g_ < *V*_1_/2) at *V*_1_ = 19 V, *V*_2_ = 0 V, and *f* = 80 Hz. Numerical simulations demonstrate that the ICEO vortex above the sinusoidal electrode could significantly enhance the transfer rate of antigen and accelerate the reaction processes within the micro cavity and thus is a valuable and rapid method to implement a higher binding efficiency, showing great potential in the time-critical disease diagnostics field.

## Figures and Tables

**Table 1:** Geometrical parameters for the structure of the optimized chip.

Parameters	Value (µm)	Implication
Lc	500	Length and width of microchannel
Hc	80	Height of microchannel
He	12.5	Height of sinusoidal electrode
Dc	125	Distance from the center of the surface reaction area to the boundary of the microchannel
Am	210	The amplitude of sinusoidal electrode
W	60	Width of sinusoidal electrode
D	25	The diameter of surface reaction area

**Table 2:** The central position coordinates of response areas in three microchannel chips.

Structure	Surface Reaction Area 1	Surface Reaction Area 2
(a)	(125 μm, 250 μm)	(375 μm, 250 μm)
(b)	(125 μm, 375 μm)	(375 μm, 125 μm)
(c)	(125 μm, 125 μm)	(375 μm, 375 μm)

## Supplementary Materials

The following supporting information can be downloaded at: https://www.mdpi.com/article/10.3390/mi13020207/s1, Figure S1: Three microfluidic chips with different positions of reaction areas. The immune reaction takes place in the circular area and the purple area is the sinusoidal electrode, Figure S2: Surface slip velocity versus grid elements, Figure S3: The plot of binding efficiency versus different inlet flow rate under *Da* = 260.04, *V*_1_ = 19 V and *f* = 80 Hz at *t* = 120 s.

## References

[1] Walls A.C., Park Y.J., Tortorici M.A., Wall A., McGuire A.T., Veesler D. (2020). Structure, Function, and Antigenicity of the SARS-CoV-2 Spike Glycoprotein. Cell.

[2] Seo G., Lee G., Kim M.J., Baek S.H., Choi M., Ku K.B., Lee C.S., Jun S., Park D., Kim H.G. (2020). Rapid Detection of COVID-19 Causative Virus (SARS-CoV-2) in Human Nasopharyngeal Swab Specimens Using Field-Effect Transistor-Based Biosensor. ACS Nano.

[3] Sigurdson M., Wang D., Meinhart C.D. (2005). Electrothermal stirring for heterogeneous immunoassays. Lab Chip.

[4] Morozov V.N., Groves S., Turell M.J., Bailey C. (2007). Three minutes-long electrophoretically assisted zeptomolar microfluidic immunoassay with magnetic-beads detection. J. Am. Chem. Soc..

[5] Sackmann E.K., Fulton A.L., Beebe D.J. (2014). The present and future role of microfluidics in biomedical research. Nature.

[6] Gervais L., de Rooij N., Delamarche E. (2011). Microfluidic chips for point-of-care immunodiagnostics. Adv. Mater..

[7] Lynn N.S., Martinez-Lopez J.I., Bockova M., Adam P., Coello V., Siller H.R., Homola J. (2014). Biosensing enhancement using passive mixing structures for microarray-based sensors. Biosens. Bioelectron..

[8] Vijayendran R.A., Motsegood K.M., Beebe D.J., Leckband D.E. (2003). Evaluation of a Three-Dimensional Micromixer in a Surface-Based Biosensor. Langmuir.

[9] Golden J.P., Floyd-Smith T.M., Mott D.R., Ligler F.S. (2007). Target delivery in a microfluidic immunosensor. Biosens. Bioelectron..

[10] Hofmann O., Voirin G., Niedermann P., Manz A. (2002). Three-Dimensional Microfluidic Confinement for Efficient Sample Delivery to Biosensor Surfaces. Application to Immunoassays on Planar Optical Waveguides. Anal. Chem..

[11] Wu Y., Ren Y., Han L., Yan Y., Jiang H. (2019). Three-dimensional paper based platform for automatically running multiple assays in a single step. Talanta.

[12] Green N.G., Morgan H. (1998). Separation of submicrometre particles using a combination of dielectrophoretic and electrohydrodynamic forces. J. Phys. D Appl. Phys..

[13] Xing X., Yobas L. (2015). Dielectrophoretic isolation of cells using 3D microelectrodes featuring castellated blocks. Analyst.

[14] Li S., Yuan Q., Morshed B.I., Ke C., Wu J., Jiang H. (2013). Dielectrophoretic responses of DNA and fluorophore in physiological solution by impedimetric characterization. Biosens. Bioelectron..

[15] Li S., Cui H., Yuan Q., Wu J., Wadhwa A., Eda S., Jiang H. (2014). AC electrokinetics-enhanced capacitive immunosensor for point-of-care serodiagnosis of infectious diseases. Biosens. Bioelectron..

[16] Wu Y.P., Ren Y.K., Tao Y., Jiang H.Y. (2017). Fluid pumping and cells separation by DC-biased traveling wave electroosmosis and dielectrophoresis. Microfluid. Nanofluidics.

[17] Wu Y., Ren Y., Jiang H. (2017). Enhanced model-based design of a high-throughput three dimensional micromixer driven by alternating-current electrothermal flow. Electrophoresis.

[18] Cui H., Cheng C., Lin X., Wu J., Chen J., Eda S., Yuan Q. (2016). Rapid and sensitive detection of small biomolecule by capacitive sensing and low field AC electrothermal effect. Sens. Actuators B Chem..

[19] Feldman H.C., Sigurdson M., Meinhart C.D. (2007). AC electrothermal enhancement of heterogeneous assays in microfluidics. Lab Chip.

[20] Selmi M., Khemiri R., Echouchene F., Belmabrouk H. (2016). Electrothermal effect on the immunoassay in a microchannel of a biosensor with asymmetrical interdigitated electrodes. Appl. Therm. Eng..

[21] Ramos A., Morgan H., Green N.G., Castellanos A. (1999). AC Electric-Field-Induced Fluid Flow in Microelectrodes. J. Colloid Interface Sci..

[22] Hart R., Lec R., Noh H.M. (2010). Enhancement of heterogeneous immunoassays using AC electroosmosis. Sens. Actuators B Chem..

[23] Wu C.C., Yang D.J. (2013). A label-free impedimetric DNA sensing chip integrated with AC electroosmotic stirring. Biosens. Bioelectron..

[24] Han D., Park J.K. (2016). Optoelectrofluidic enhanced immunoreaction based on optically-induced dynamic AC electroosmosis. Lab Chip.

[25] Han D., Park J.K. (2016). Microarray-integrated optoelectrofluidic immunoassay system. Biomicrofluidics.

[26] Haab B.B. (2005). Antibody arrays in cancer research. Mol. Cell. Proteom..

[27] Anand R.K., Johnson E.S., Chiu D.T. (2015). Negative dielectrophoretic capture and repulsion of single cells at a bipolar electrode: The impact of faradaic ion enrichment and depletion. J. Am. Chem. Soc..

[28] Morales-Narvaez E., Guix M., Medina-Sanchez M., Mayorga-Martinez C.C., Merkoci A. (2014). Micromotor enhanced microarray technology for protein detection. Small.

[29] Ge Z., Yan H., Liu W., Song C., Xue R., Ren Y. (2020). A Numerical Investigation of Enhancing Microfluidic Heterogeneous Immunoassay on Bipolar Electrodes Driven by Induced-Charge Electroosmosis in Rotating Electric Fields. Micromachines.

[30] Gonzalez A., Ramos A., Green N.G., Castellanos A., Morgan H. (2000). Fluid flow induced by nonuniform ac electric fields in electrolytes on microelectrodes. II. A linear double-layer analysis. Phys. Rev. E Stat. Phys. Plasmas. Fluids. Relat. Interdiscip. Top..

[31] Wu Y., Ren Y., Tao Y., Hou L., Jiang H. (2018). High-Throughput Separation, Trapping, and Manipulation of Single Cells and Particles by Combined Dielectrophoresis at a Bipolar Electrode Array. Anal. Chem..

[32] Wu Y., Ren Y., Tao Y., Hou L., Hu Q., Jiang H. (2016). A novel micromixer based on the alternating current-flow field effect transistor. Lab Chip.

[33] Wu Y., Chattaraj R., Ren Y., Jiang H., Lee D. (2021). Label-Free Multitarget Separation of Particles and Cells under Flow Using Acoustic, Electrophoretic, and Hydrodynamic Forces. Anal. Chem..

[34] Wu Y., Hu B., Ma X., Zhang H., Wang Y., Li W., Wang S. (2022). Generation of droplets with adjustable chemical concentrations based on fixed potential induced-charge electro-osmosis. Lab Chip.

[35] Squires T.M. (2009). Induced-charge electrokinetics: Fundamental challenges and opportunities. Lab Chip.

[36] Harnett C.K., Templeton J., Dunphy-Guzman K.A., Senousy Y.M., Kanouff M.P. (2008). Model based design of a microfluidic mixer driven by induced charge electroosmosis. Lab Chip.

[37] Wu Y., Ren Y., Tao Y., Hou L., Jiang H. (2016). Large-Scale Single Particle and Cell Trapping based on Rotating Electric Field Induced-Charge Electroosmosis. Anal. Chem..

[38] Kim H.Y., Lee J.H., Kim M.J., Park S.C., Choi M., Lee W., Ku K.B., Kim B.T., Park E.C., Kim H.G. (2021). Development of a SARS-CoV-2-specific biosensor for antigen detection using scFv-Fc fusion proteins. Biosens. Bioelectron..

[39] Wang B., Cheng X. (2016). Enhancement of binding kinetics on affinity substrates by laser point heating induced transport. Analyst.

[40] Lynn N.S., Homola J. (2015). Biosensor enhancement using grooved micromixers: Part I, numerical studies. Anal. Chem..

