# Supplementary material for: Enhancement of Binding Kinetics on Affinity Substrates Using Asymmetric Electroosmotic Flow on a Sinusoidal Bipolar Electrode
Source: Micromachines (Basel). 2022 Jan 28;13(2):207. doi: 10.3390/mi13020207 (PMC8878551; doi:10.3390/mi13020207)
Supplement: Supplementary file 1 [file micromachines-13-00207-s001.zip › micromachines-1557323-supplementary.pdf]

Enhancement of binding kinetics on affinity substrates using asymmetric electroosmotic flow on a sinusoidal bipolar electrode.

Yupan Wu[123], Bowen Hu[1], Xun Ma[1], Yucheng Wang[1], Wei Li[1] and Shaoxi Wang*[1]

[1] School of Microelectronics, Northwestern Polytechnical University, Xi'an, PR China 710072, Email:
[2] Research & Development Institute of Northwestern Polytechnical University in Shenzhen, PR China 518000
[3] Yangtze River Delta Research Institute of NPU, Taicang, PR China 215400
* Correspondence: shxwang@nwpu.edu.cn

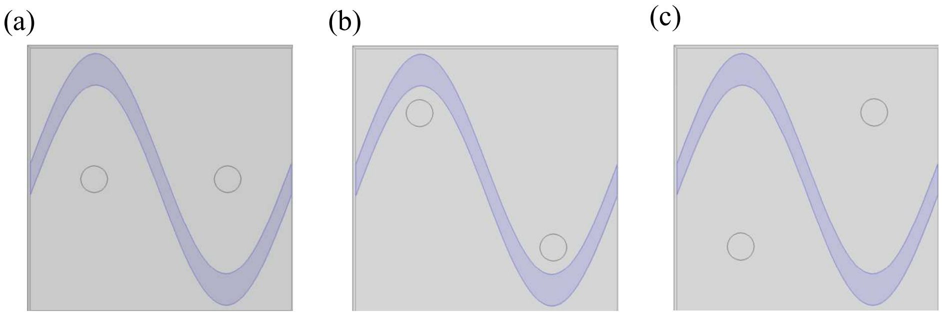

Figure S1. Three microfluidic chips with different positions of reaction areas. The immune reaction takes place in the circular area and the purple area is the sinusoidal electrode.

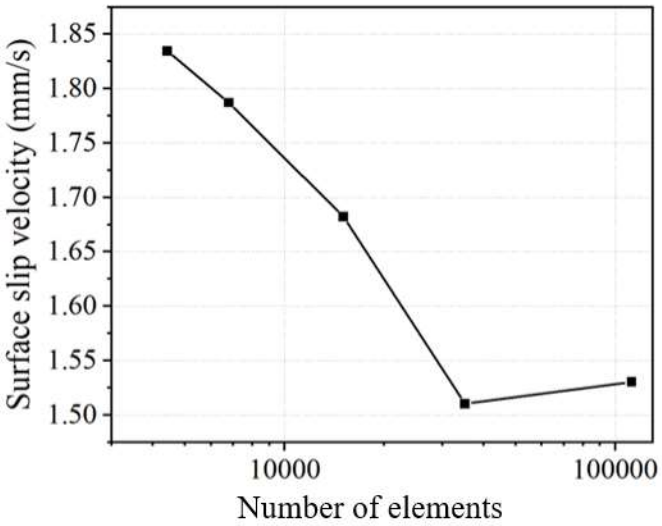

Figure S2. Surface slip velocity versus grid elements.

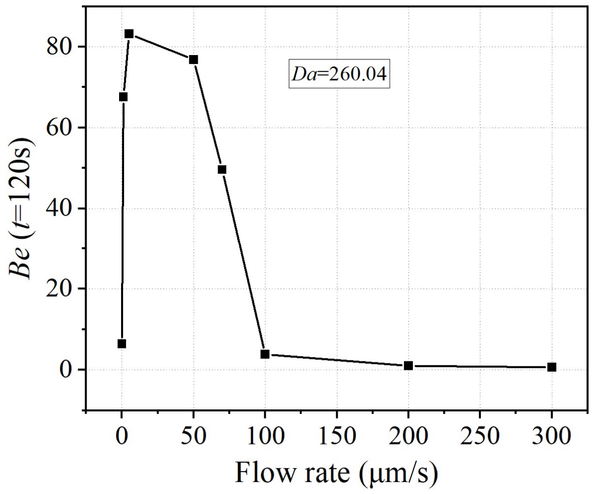

Figure S3. The plot of binding efficiency versus different inlet flow rate under *Da*=260.04, V_1=19V and *f*=80Hz at *t*=120s.
